# 

**Published:** 1873-11

**Authors:** 


					CONTENTS:-
ORIGINAL COMMUNICATIONS.
-Pivot Teeth.
By E. D. Swain, D. D. S.
24
Article J.?
-May the Calcific Elements of the Deciduous Teeth be Appropriated in the
Formation oi any portion or the Permanent Ones?
By M. Si Dean,
D. V. S.
2f
Abticle II.-
?Vulcanite Litigation.
By Samuel S. White, D. D. S.
*
Article III.
SELECTED ARTICLES.
-Yital Actions which Play an Important Part in Dental Caries.
By Henry
S. Chase, M. D., D. D. S.
310
Article IV -
-Swallowing Artificial Teeth.?Death and Autopsy.
By E. D. Smith M. D.
3%
Article v.-
?Gelseminum in Odontalgia.
By J. W. Legg, M. D.
31
Article VI.-
-Amalgam Fillings.
By Thos. Fletcher, F. 0. S..
32
Article VII.-
-Digestive Power of baliva and Pancreatic Juice during infancy.
3d
Article Yiil.-
-When to Lance the Gums.
By J. L. Smith, M. D.
33
Abticle IX.-
?Diarrhoea of Teething Children.
By W. H. Day, M. D
32t
Article X.-
-Treatment of Morbid Dentition.
By E. D. Drake, M. D.
32
Article XI.-
EDITORIAL, ETC.
Dental Education
Action of Nitrous Oxide
Roughening Plugger Points.
MONTHLY SUMMARY.
33
A Rare and Extraordinary Case...
Treatment of Salivation by Atropia
Hypertrophy of the Tongue....!.......
Poisonous Character of Nitrous Oxide
Ice in the Treatment of Facial Neuralgia....
Bleaching Wax.....
Ointment for Neuralgia
Remarks on Methylene
Treatment of Burns
33
33
33
33
33!
33!
331
331
OBITUARY.
Cyras S. Knapp,
33(1
D. D. S.

				

